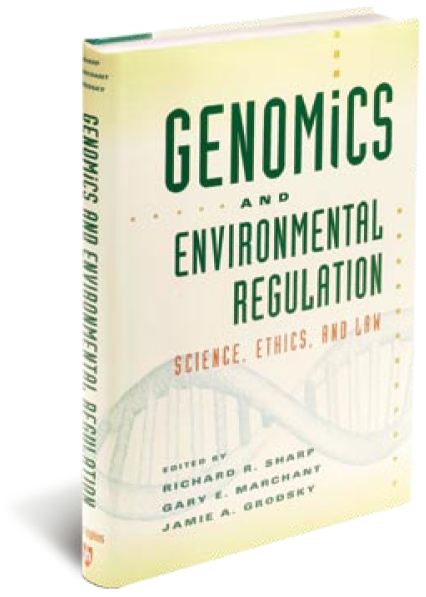# Genomics and Environmental Regulation: Science, Ethics and Law

**Published:** 2009-09

**Authors:** Raymond W. Tennant

**Affiliations:** *Raymond W. Tennant began his career at the Oak Ridge National Laboratory, and has been chief of the Genetic Toxicology Branch, chief of the Laboratory of Environmental Carcinogenesis, and director of the National Center for Toxicogenomics at NIEHS.*

*Genomics and Environmental Regulation* is a canonical compilation of fundamental issues of this topic by leaders in the field. This timely work focuses on the potential and problems arising from the application of genomic technologies to toxicology and environmental policy, and is divided into four parts—“Environmental Policy Perspectives,” “Legal Perspectives,” “Occupational Health Perspectives,” and “Ethical and Philosophical Perspectives”—each containing several chapters by the editors and other leading scholars.

What makes these topics of such interest and complexity is that scientists can perceive the potential value of genomic technologies that have not yet been developed sufficiently to be applied. Ethicists and the legal system perceive both the potential value and the challenges associated with the applications of genomic technology. This compilation is a state-of-the-art assessment of the ethical and legal implications of using data derived from genomics to address issues related to environmental and occupational exposures, including use by regulatory agencies and in the courtroom.

In the first chapter, Gary Marchant focuses on toxicogenomics and explores the many applications, challenges, and limitations in using data from a discipline that is in a dynamic state of development. Next, Dearfield et al. describe the current strategy of the U.S. EPA in attempting to create a framework for using genomics technologies. Richard Phillips describes studies on predictive toxicology and understanding mechanisms and modes of action in risk assessment; he provides insights into the actual state of development of toxicogenomics as well as issues related to genetic susceptibility. John Balbus deals with technical and sociopolitical challenges to the implementation of toxicogenomics, identifying how toxicogenomics may be used to address the limitations of conventional toxicological testing.

In “Legal Perspectives,” Lynn Burgeson discusses challenges in applying toxicogenomic data in federal regulatory decisions. She discusses genomic data, information quality, and peer review in the context of agency actions in adverse effects reporting. Askland and Marchant discuss legal complications of the combination of polymorphisms and occupational or environmental exposure that together may increase the risk of illness—specifically the use of genetic data in toxic tort suits relating to the causation of specific illnesses. They also discuss the the possibility that genomic data may have discriminatory impacts. Marchant and Grodsky address the disparate impact of environmental exposures on different communities and the possible role of genetic variations. And Marchant discusses the setting of national ambient air quality standards (NAAQS), perhaps one of the first applications of genetic susceptibility data. He believes that the NAAQS are among the nation’s most important environmental regulations because they provide the greatest health benefits and largest compliance costs. The chapter comprehensively discusses the NAAQS program, genetic susceptibility to criteria air pollutants, normative issues, and alternative approaches.

In Part 3, Paul Schulte covers workplace issues and occupational health and the potential uses and challenges arising from genetic testing and surveillance using genomic technologies. Marc Weinstein extends the discussion to the use of genomics in epidemiology and its implications for occupational health and disease prevention. Mark A. Rothstein deals with occupational and health issues raised by the use of toxicogenomics in the workplace, including the Occupational Safety and Health Act, Americans with Disabilities Act, genetic testing and monitoring, and nondiscrimination laws. Kenneth Mossman discusses genetic susceptibility and radiological health and safety.

Part 4 contains chapters by by Smith and Robert on “Conceptual and Normative Dimensions of Toxicogenomics” which presents a skeptical view point on the informational content and ultimate utility of toxicogenomics and toxicogenetics; by Resnik on “Environmental Disease, Biomarkers, and the Precautionary Principle”; by Nickel on “Rights and the Exceptionally Vulnerable”; by Cranor on “(Almost) Equal Protection for Genetically Susceptible Subpopulations – A Hybrid Regulatory – Compensation Proposal”; and by Finkel on “Protecting People in Spite of – or Thanks to – The Veil of Ignorance.”

The book’s appendix presents the 2007 report by the National Academy of Sciences on “Applications of Toxicogenomic Technologies to Predictive Toxicology and Risk Assessment” and the book is adequately indexed. The preface nicely presents a statement of the goals of the book and a concise synopsis of each chapter. The book will be of particular value to those whose interests bridge the technical and legal–ethical issues at the forefront of toxicology today.

## Figures and Tables

**Figure:**